# Being alive to the world: an artist's perspective on predictive processing

**DOI:** 10.1098/rstb.2022.0429

**Published:** 2024-01-29

**Authors:** Robert Pepperell

**Affiliations:** Cardiff Metropolitan University, Cardiff CF5 2YB, UK

**Keywords:** ambiguity, indeterminacy, paradox, predictive processing, art, visual space

## Abstract

I consider predictive processing (PP) from the perspective of an artist who also conducts scientific research into art and perception. This paper presents artworks I have made and statements from other artists that exemplify some of PP's core principles. But it also raises questions about the extent to which current applications of PP theory provide a comprehensive account of art experience. Prediction error minimization, a key mechanism of PP, has been proposed as a cause of positive aesthetic affect because artworks offer opportunities for reward through disambiguation and learning. However, there are many cases where prediction errors proliferate in art experiences in a way that enhances aesthetic affect. Here I suggest the inability of our perceptual systems to minimize prediction errors when beholding certain artworks can evoke heightened states of fascination and exhilaration. Moreover, powerful artworks provide opportunities for maximizing prediction errors, within certain bounds, by evoking states of paradox, contradiction and illogicality. I conclude that beholding such artworks can intensify our sense of being by making us more alive to the world.

This article is part of the theme issue ‘Art, aesthetics and predictive processing: theoretical and empirical perspectives’.

## 1. Introduction

I am an artist who makes work that addresses questions about the nature of the mind and its relationship to the world. I also use scientific methods and knowledge to understand the nature of art, which in turn informs my attempts to better understand mind and reality. In this opinion piece I reflect on how the predictive processing, or PP, approach to perception and action, which is now being applied in many areas of science and philosophy, can contribute to our understanding of art experience [1–3].

For anyone unfamiliar with its history and theory, PP can seem to confound some of our most basic intuitions about perception. When we look at a painting, let us say, we see an artefact that appears to be vividly present in the world, composed of detailed forms, textures and colours, and depicting objects imaginary or real. But according to PP, what we see is not what is in the world or even what is given in our sensory data, which are necessarily noisy and ambiguous. Rather, what we see is created by the brain as it hierarchically generates probabilistic predictions about the most likely causes of the sensory data, given that it has no direct access to those causes. As some PP theorists put it, what we really perceive are the brain's ‘best guesses’—or inferences—about what is in the world using current sensory data and knowledge derived from prior experience [4].

This general approach has many implications for the way we think about art experience, both in terms of how art is perceived and how it is made, as this theme issue exemplifies. I will not provide a detailed account of PP theory; such accounts can be found elsewhere in this issue. Rather I will consider some of the implications that, from an artistic perspective, seem to be especially salient and, from a scientific perspective, seem to have explanatory promise. I will do so with reference to examples from my own art practice and the works and writings of other artists. My aim is to explore how the PP approach helps us to understand some of the perceptual and aesthetic effects evoked by works of art, and why they bring value to our lives.

## 2. Visual indeterminacy in artworks

Art history furnishes us with many examples of acclaimed artworks that evoke powerful affective responses even though they resist easy or immediate identification, a phenomenon I have called ‘visual indeterminacy’ [5]. Such works can be full of detailed forms and highly suggestive object cues, but do not allow for any definite interpretation. Historical examples in the European and American traditions include the late paintings of JMW Turner and many works of Impressionism, Cubism and Abstract Expressionism. A contemporary example is the British painter Cecily Brown, who cites her interest in Gestalt theories of perception and the indeterminate paintings of the post-impressionist painter Edouard Vuillard, saying: ‘I'm obsessed with the suggestiveness of the marks and the way the brain responds: how you see things that you're not sure you're seeing, and that kind of uncanny and hallucinatory quality of everyday life’ [6]. Interestingly, the emotional reactions of the public, and even of other artists, to indeterminate artworks has not always been positive—initially at least [7]. Such was the hostility to Impressionism at its first exhibition in Paris in 1874 that people reportedly spat at the paintings in disgust [8].

**Figure 1.**
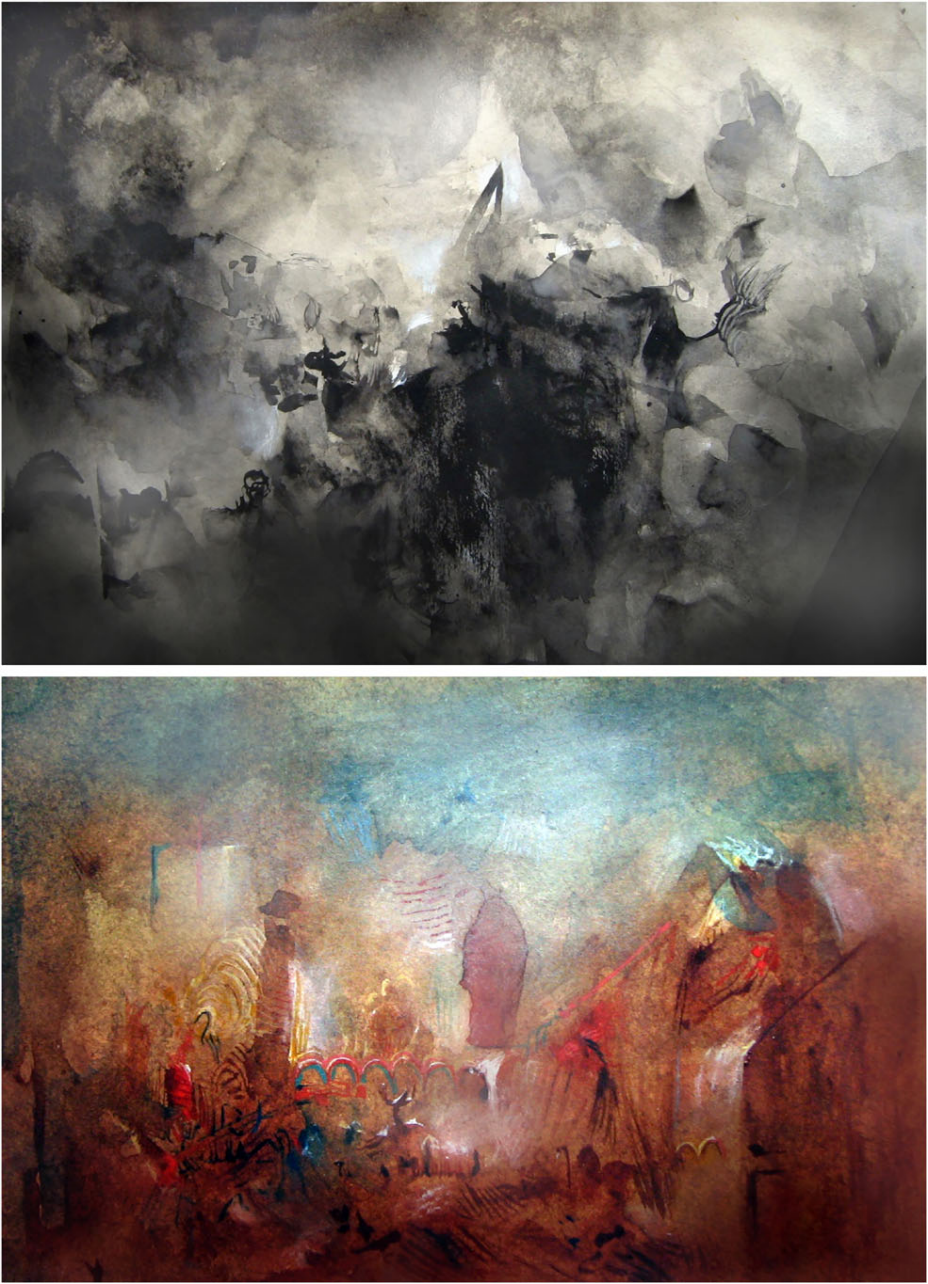
Two examples of visually indeterminate artworks made by the author: (upper) *We Will Never Forge*t, watercolour on paper, 10 × 15 cm, 2007, and (lower) *The Wonderful Machine*, watercolour on paper, 2007, 10 × 17 cm. Both these works encourage the viewer to look for objects or familiar forms that might help to identify their subject matter. In doing so, according to PP, the viewer tries to infer what the paintings depict based on the sensory data by generating and, where necessary, updating various perceptual hypotheses.

A personal encounter with visual indeterminacy when I was a student—described in Pepperell [5]—inspired the creation of a large body of artworks that aimed to evoke the same sense of perceptual uncertainty in other people that I had briefly experienced myself. In fact, the longer one studies these works in person the more possible meanings seem to suggest themselves, yet the more certain one becomes that they have no specific meaning at all. The development of this work and related scientific studies are described elsewhere [9,10], and examples can be seen in figure 1. Here I consider these works from a PP perspective.

One of the challenges in developing the technique used to make these works was the need to satisfy a version of what is known as the Goldilocks effect, the finding from psychology that human infants prefer stimuli having the optimal balance of complexity and simplicity [11]. To evoke the visually indeterminate state it was necessary to get the balance between abstraction and representation ‘just right’, which I as the artist had to learn—by trial and error—to optimize in my own perceptual experience. If the pictures were too abstract then I and the viewers simply categorized them as textures; if the forms were too suggestive of, say, bodies or buildings then there was no incentive to search further for meaning. After several years of experimentation with different techniques I was able to tell from viewers' body language and facial expressions when I was on the right track; viewers who reported experiencing visual indeterminacy tended to momentarily freeze in front of the work with a look of mild surprise.

The PP approach might explain the perceptual effects evoked by these artworks as follows. The indeterminate artwork presents a rich source of bottom-up sensory data consisting of forms, colours, contrasts and textures that need to be accounted for by the viewer's top-down perceptual processes, including my own as the artist. These processes attempt to predict, using an approximate form of probabilistic reasoning, what the sensory data is most likely to represent in the world, given that this cannot be known directly. Where there is a mismatch between these predictions and the data, a prediction error is generated. This prompts the perceptual system to update its model so that, ideally, it matches the sensory data. Normally, when we are confronted with a detailed rectangular arrangement of colours, textures and shapes we might have a high expectation of recognizing objects in it—an expectation forged by a lifetime of looking at recognizable pictures. But indeterminate images tend to thwart this process because the hypotheses generated to account for the sensory data will never be satisfactorily confirmed. Consequently, prediction errors continue to propagate through the system as long as the viewer attends to the work.

Several proposals have been made by PP theorists to account for the aesthetic power and affective properties of indeterminate or ambiguous artworks. Van de Cruys & Wagemans [12], for example, suggest reward is gained when transitioning from a state of uncertainty to one of greater predictability as viewers recover meanings that the artist has suggested but withheld. In a similar vein, Frascaroli [13] points to the inherent pleasure of problem solving, as is sometimes experienced in the ‘Aha’ moment of insight [14]. But he also argues that some stimuli offer more opportunities than others for tuning our internally generated model to the unpredictable contingencies of the external world.

The perceptual challenges afforded by ambiguous artworks can be one such case, and he quotes one of the leading figures of the PP approach, Karl Friston, who states that inferential acts are most pleasurable not when when placing ‘safe bets’ but when ‘disambiguating between plausible and competing hypotheses' [15]. Van de Cruys and colleagues [16] make the related argument that the pleasure or positive valence to be gained from ambiguous artworks lies in the opportunities they afford for increasing the rate of prediction error minimization rather than in removing such errors *per se*. A similar point is made by Seth [4] in his paper linking predictive processing to the notion of the ‘beholder's share’ in artistic experience, a term popularised by the art historian Gombrich [17]. Seth cites the work of Joffily and Coricelli [18] who argue that sensations which increasingly violate expectations produce negative emotional valence, while those that increasingly match expectations produce positive emotional valence. This provides the perceiving agent with constant affective feedback which signals how well it is doing in regulating homeostasis and adapting to the uncertainties of its environment. These and other related suggestions may help, in part, to explain the complex aesthetic experiences associated with ambiguous and indeterminate artworks using fundamental principles from cognitive neuroscience.

There have been recent attempts to empirically quantify the multiple divergent associations evoked by indeterminate images, and their effect on aesthetic judgements. Pablo Picasso once said about a work of art that ‘the more associations it can open up the better’. [19] Prompted by this observation, colleagues and I recently conducted a study using computational methods which found that ambiguous or indeterminate images tended to elicit more numerous and diverse textual descriptions than recognizable images [20]. A follow-up study found a positive correlation between this measure and aesthetic ratings of ambiguous images. But this was so only for approximately half of the participants; the other half preferred more recognizable images with fewer and less diverse descriptions [21].

This finding raises a question, in my mind at least, about how these preferences for multiplicity and heterogeneity of meaning, and their variance among the population, could be accommodated within the PP approach; why is it, for example, that an ambiguous image that generates multiple and diverse hypotheses, none of which is finally confirmed, is more aesthetically attractive to some people, given that there is no prospect of reward from uncertainty reduction? I will return to this question below.

## 3. Dichotomy and twofoldness in picture perception

**Figure 2.**
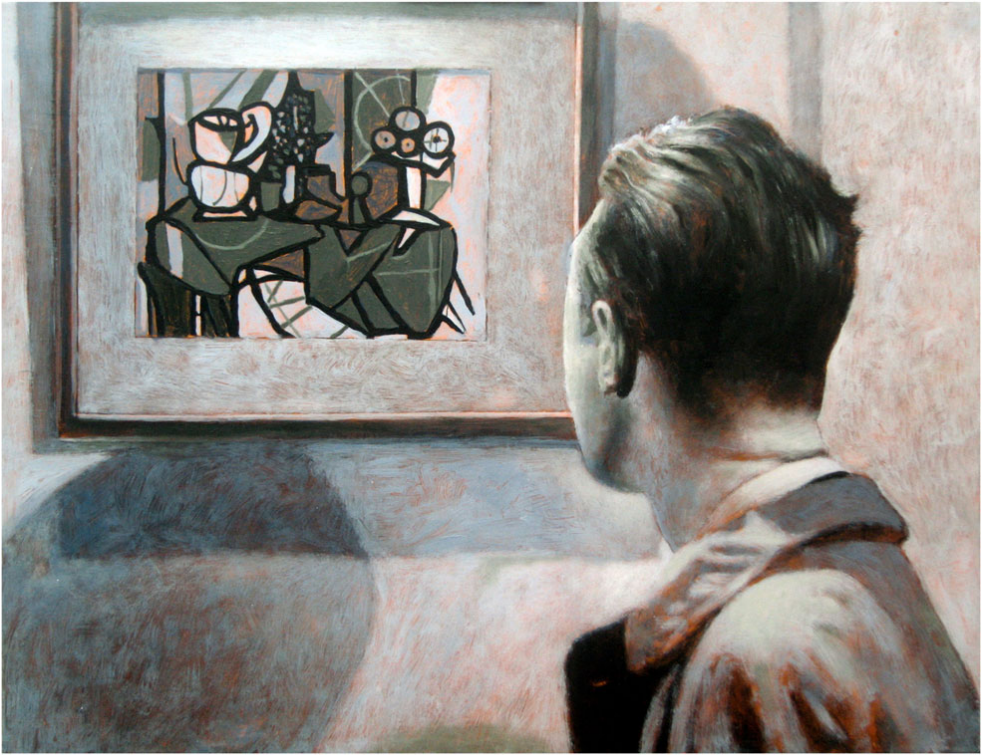
*Policeman Bemused by Picasso*, oil on panel, 2009, 40 × 60 cm. This painting by the author depicts the moment in Alfred Hitchcock's movie *Suspicion* [22] when a policeman sees a reproduction of Picasso's *Pitcher and Bowl of Fruit* [23]. During this brief scene the audience is given to understand that the beholder is uncertain about what he sees, as presumably they would have been if they were unfamiliar with Picasso's recent work.

The painting reproduced in figure 2 is one of a series on the theme of visual indeterminacy in cinema that I made in the late 2000s. It is based on a still from the movie *Suspicion*, directed by Alfred Hitchcock [22], and shows the moment when a policeman is briefly bemused by a reproduction of a Picasso painting that he finds hanging on the wall of a chic residence. The policeman's bodily behaviour—which echoes that I often witnessed in the viewers of my own indeterminate works—makes clear to the audience that this object violates his expectations about what a picture hanging on a wall should be.

*Policeman Bemused by Picasso* is shown here to illustrate a point, not about visual indeterminacy as such but about the nature of all pictorial representation. Pictures, as the philosopher Richard Wollheim argued in a famous essay, have a ‘twofold’ character in that they present us with two distinct, even conflicting, aspects at the same time [24]. When we view a picture, we are aware of seeing both the material from which it is made and the content it represents. In the case of viewing *Policeman Bemused by Picasso* in person*,* you see both a man looking at a painting and a panel of wood covered in brushed paint.

Elsewhere I have described this twofold aspect of pictures as a dichotomy because of the seemingly impossible position it puts us in as perceivers [25]. Indeed, one of the most prominent advocates of the ‘perception as inference’ approach, Richard Gregory, said that pictures are ‘impossible’ for exactly this reason, and that the incompatibility between these ‘double realities’ constitutes a ‘paradox’ [26]. Wollheim's original argument has generated voluminous debate among philosophers of art and aesthetics, including a famous dispute between him and Gombrich about whether we see these two aspects simultaneously or alternately (see [27]).

The twofold or dichotomous nature of picture perception is a fundamental feature of many art experiences and is often mentioned by artists as one of the reasons that art can engage us so powerfully. In an interview given in 1960, for example, the painter Philip Guston said: ‘In those great Rembrandts there's an ambiguity of paint being image and image being paint, which is very mysterious' [28, p. 14]. Yet to the best of my knowledge, it has not been explicitly considered in the context of the PP framework (see footnote 12 in [29]). From a PP perspective, the dichotomy might be explained because the sensory data presented by the picture generates two plausible but conflicting hypotheses at once. When viewing the original version of the painting reproduced in figure 2, you perceive both a man looking at a painting and a rectangular surface covered in patches and marks of paint. The impossibility of the dichotomy rests on the inability of our perceptual processes to adjudicate between the conflicting hypotheses; what reason would it have to favour one over the other?

This raises a question about how the PP approach can explain this dichotomous effect. Because of the persistence of this dichotomy—and indeed its intensification as viewing time increases—the aesthetic effect of pictures cannot be attributed to the reward gained from uncertainty reduction. Nor can it be accounted for by the pleasures of disambiguating among competing hypotheses as suggested by Karl Friston above. And given that prolonged viewing of a picture increasingly violates the strong expectation (or what has been called a Bayesian ‘hyperprior’ [2]) that a perceived object should be either one thing or another, and not two different things at the same time, the valence of pictorial experience should be negative according to the theory of Joffily & Coricelli [18]. Nevertheless, our experiences of pictures *per se* are not generally unpleasant, and in fact prolonged or ‘slow viewing’ of an ambiguous work of art is more likely to enhance aesthetic experience [30].

An alternative way to account for this phenomenon, which I will return to below, is that the very impossibility of resolving the dichotomy between the competing hypotheses produces a state of ‘perceptual dissonance’ in the beholder. In its mildest form this evokes interest and in its strongest form it evokes a sense of fascination, even exhilaration, that is intrinsically exciting or arousing [25,31]. Painters are often aware of this, and deliberately exploit it for aesthetic effect. In *Policeman Bemused by Picasso*, for example, the dichotomy is heightened by the saliency of the paint handling, which is more apparent when viewed in person. The original Picasso painting itself achieves the same ends in a different way. He describes ordinary household objects with an abstruse graphical style that foregrounds its own artifice.

## 4. Looking at depictions of visual space

**Figure 3.**
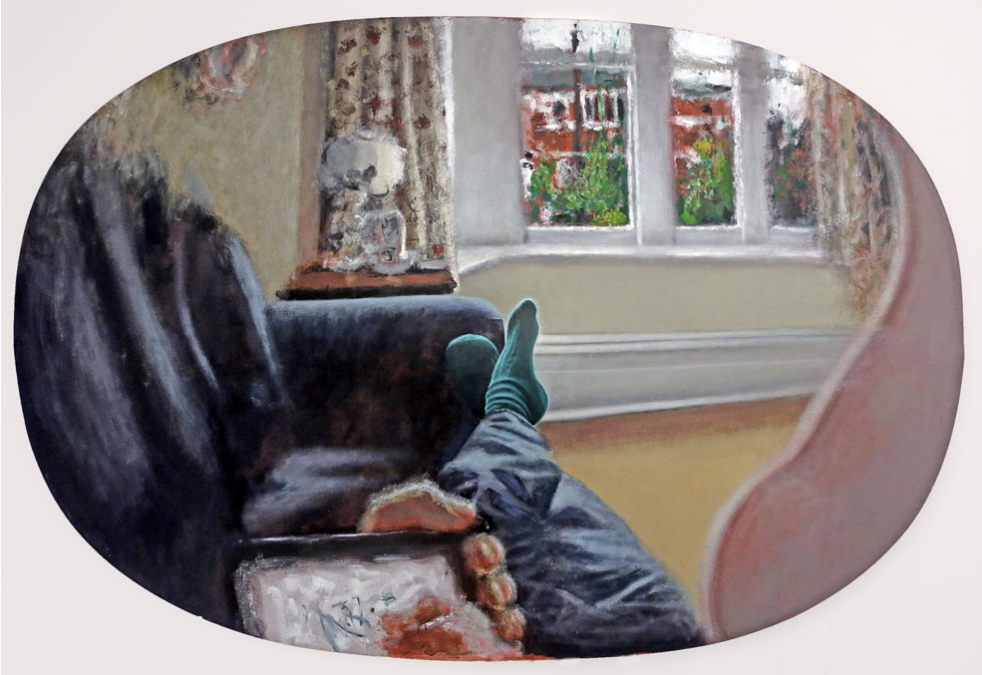
*Self-portrait (after Mach)*, oil on formed canvas, 2012, 100 × 150 cm. This painting is one of a series in which I record the structure of my visual field as accurately as possible when fixating on a point in space, in this case my own feet. This is a monocular view, showing the visual field of my left eye, and including the entire contents of my peripheral field, with my nose on the right.

The painting reproduced in figure 3 presents a different kind of pictorial dichotomy, one that can only be fully appreciated when the original is viewed in person, at length and, as will be discussed below, in a particular way. It is one of a series of works in which I tried to paint and draw the structure of my visual field, including the entire periphery, when fixating on a point in space before me. In most cases these works show my binocular viewpoint, but *Self Portrait (after Mach)* depicts what I saw with my left eye when looking at my feet. Elsewhere I have written about the making of these works, which are products of intense scrutiny of my visual phenomenology, and their relevance to art history and perception science ([32]; see also Seth [4] for a discussion of this work in PP terms). Here I focus on how they are viewed and the implications for another important element of the PP approach, namely the crucial role of action in prediction error minimization, or what is known in PP terms as active inference.

**Figure 4.**
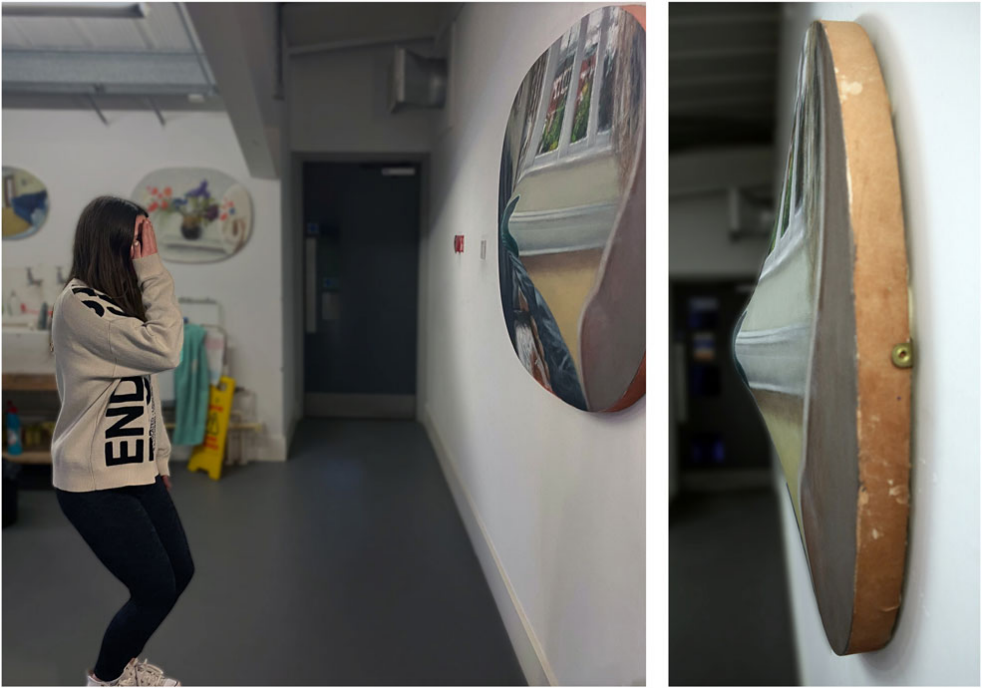
The image on the left shows a beholder adopting the ideal viewing position for *Self-portrait (after Mach).* She is viewing it with her left eye, which is located at the centre of projection of the painting and in line with the green socks. Viewed from this position she sees approximately the same patterns of light that I saw and recorded in the painting. This produces a feeling of depth and immersion, and a sense that the body parts in the painting are part of the beholder's body. The image on the right shows the convex bulge in the painting and the matte paint that has been applied anamorphically to conceal it when the painting is viewed head on. If the beholder moves slightly when viewing in the ideal position, they perceive a subtle motion parallax between the green socks and the wall behind.

*Self Portrait (after Mach)* is most aesthetically potent when the beholder adopts a specific viewing position in front of the original work. Because the painting is, in effect, a geometrical projection of the three-dimensional space I perceived, like the two-dimensional projection of space created by the rules of linear perspective, it has a specific ‘centre of projection’ [33]. This is a point in front of the painting that, when occupied by the single eye of the beholder looking at the painted feet from the position illustrated in figure 4 (left) causes the pattern of light reflected from the painted surface to emulate that I experienced when I recorded the scene. Viewing the painting in this way can produce a surprising sense of realism or immersion, a little like that experienced in virtual reality, while the body parts shown in the picture can appear to be extensions of the beholder's own self. At the same time, of course, the beholder remains fully aware of the painted canvas surface and hence the artifice of the illusory space.

In this work, the prediction errors generated by the divergence between bottom-up sensations and top-down predictions are more pronounced than would be the case with viewing a conventional linear perspective depiction of visual space. Most linear perspective pictures show only the central region of the visual field, cropping the peripheral field, and are normally viewed with two eyes from outside the centre of projection. When *Self Portrait (after Mach)* is viewed in the way prescribed there is an unusually close correspondence between the visual field data provided by the painting and the spatial structure we are used to experiencing in natural vision, including those parts of our own body often seen when reclining on a sofa. This increases the beholder's confidence that their sensory data is consistent with seeing the contents of their own visual field before them. But this hypothesis is nevertheless contradicted by the evident painterly properties of the picture surface, which prevents minimizing the resulting prediction error. This surprising coincidence of strong sensory evidence and high prediction errors seems to be associated with feelings of fascination and exhilaration that, as noted above, can characterize certain art experiences.

What cannot be seen in figure 3, however, is that the physical surface of *Self Portrait (after Mach)* has a pronounced convex bulge in the region around the feet, as shown in figure 4 (right). This is not apparent when the original painting is approached from the front since the bulge is concealed by anamorphic application of matte paint [34]. But when the beholder adopts the viewing attitude described above and then moves slightly to the left or right, or up or down, they experience a subtle motion parallax in the painting between the green socks and the wall behind. This induces an even more surprising illusion of depth in the picture as a whole and an ever-stronger sensation that one is seeing one's own (virtual) body in one's (virtual) visual field.

The imaginary motion parallax is generated by visual processes occurring in the brain as it attempts to reconcile various ambiguous depth cues with a lifetime's experience of actual motion parallax (see [35] for a discussion of the ‘paradoxical pseudo-parallax’ evoked by these paintings). These ambiguous depth cues include the convex bulge of the painting which is perceived as extending towards the beholder in physical space, the illusory depth rendered in paint, which is perceived as receding away, and the unexpected deformation of the painted surface as the beholder moves. The ambivalence of these cues tends to increase rather than diminish the longer the painting is viewed in the suggested way.

A similar effect is powerfully demonstrated by the contemporary artist Patrick Hughes, who is widely known for the ‘reverspective’ paintings he has been making for many decades. These consist of protruding surfaces that—as the beholder moves—seem to contradict the spatially recessive perspective cues painted onto them, in much the same way as *Self Portrait (after Mach)*. He says: ‘I think my reverspectives give this sense of continual flux and reciprocity; as they move you move, and what is more they move in the opposite way to what you expect, which is why one says they move although they are solid and immovable.’ [36, p. 7].

These cases illustrate the way in which our embodied interaction with an artwork can strongly affect our perception of it, to the extent that pictures on flat or deformed surfaces can cause us to see illusory depth and motion parallax without losing our awareness of the picture surface itself—even when the perceived direction of the deformation directly contradicts that of the illusion. With colleagues Alistair Burleigh, Nicole Ruta and others, I continue to investigate these and similar effects in the fields of perception science and computer graphics [37,38]. For present purposes, I note that these examples illustrate a further key theoretical principle of the PP approach.

A principal feature of PP, as propounded by theorists such as Seth [4], Clark [39], Howhy [1] and Friston [3], is that the biological purpose of perception is to guide effective action. In fact, perception and action are two aspects of one continuous process through which we navigate the world in ways that keep us within states that are optimal for survival. Given that the causes of sensory data are inaccessible to an agent and must be inferred using hierarchical probabilistic predictive processes, the agent can improve the quality of the evidence on which its inferences are based by changing its sensory data through action, that is, by moving with respect to the world.

Some artworks, as noted here, encourage such active exploration. Viewing *Self Portrait (after Mach)* in the way described is one example of active inference at work. By adjusting their position with respect to the painted surface, beholders can continually acquire new sensory evidence, and the longer and more actively they look the more evidence they can acquire. But in cases where active exploration proliferates conflicting or contradictory evidence, as with the artworks discussed in this section, prediction errors and therefore uncertainties increase with the surprising result that aesthetic affect in the beholder can also increase. In the remainder of this paper, I offer a suggestion for how this and other related aspects of art experience might be accommodated within the PP framework.

## 5. Opportunities and questions

This issue of *Phil. Trans. R. Soc. B* exemplifies the growing trend in which scientists apply their tools and theories to the study of art, not only to learn what science can discover about art but also what art can teach science. My own experience over many years has taught me that integrating art and science research such that both contribute and benefit equally can be highly challenging, not least because of the deep-seated differences in history and mindset between these fields. In writing this opinion piece I have found a surprising degree of commonality between the ideas that motivate many artists and the core principles of the PP approach. These commonalities present an opportunity for genuine art–science dialogue through which new knowledge could be generated that neither could generate alone.

Given that we are in the early stages of capitalizing on this opportunity, questions inevitably remain that need to be considered if the PP approach is to provide a comprehensive account of art experiences. I will briefly mention three. The first is that much of the literature generated by scientific studies of art conflates aesthetic affect with pleasure. There is little doubt that art experiences can be pleasurable, as statements by artists confirm, and perhaps this has led some to think that they are *only* pleasurable, or even worse, merely pleasant. As noted at the outset, art experiences are complex and powerful and can even include negative feelings such as disgust. So, while the pleasures and rewards of art experience need to be accounted for, so do other aesthetic effects that I have suggested here form part of those experiences, including bemusement, surprise, fascination and exhilaration. Recent work is beginning to address this [16,40].

A second related issue has been touched on already in the discussion of pictorial dichotomy and concerns a sentiment that frequently pervades artistic discourse, especially in the modernist tradition. It is that art experience, especially when at its most powerful, often entails paradoxical, contradictory, and illogical states of mind. Patrick Hughes said: ‘I embrace the contradictory and celebrate the paradoxical. A paradox to me is like a pearl’ [36, p. 7]; the conceptual artist Sol LeWitt said: ‘Conceptual art is not necessarily logical’ [41, p. 80]; and Bridget Riley talks of her: ‘deeper involvement with the structure of contradiction and paradox in my more recent work’ [42, p. 143]. This cultivation of dissonant mental states, such as those evoked by pronounced pictorial dichotomy, may bear on the sense of mystery that is often cited by artists as the hallmark of intense aesthetic experience—the surrealist painter René Magritte said: ‘The feeling, the picture and we ourselves are united in our mystery.’ [43, p. 74].

The third issue is that mentioned above in respect to the quote from Picasso, which is the way artworks can give rise to multiple and diverse associations and which is expressed in the preference some people show for indeterminate or ambiguous images. This also suggests that other motives may be at work in addition to those driving a perceiving agent towards uncertainty reduction or maximizing opportunities for learning.

## 6. Being alive to the world

We can begin to accommodate these important aspects of art experience within the PP framework, I suggest, by learning from the many artists who have investigated the relationship between art, perception, and aesthetics. Artists often talk insightfully about the power and fallibility of perception, and about how we can fine-tune our perceptual capacities over time. The painter Sargy Mann, for example, was extraordinary in several ways, not least because he continued to paint successfully as he gradually lost his sight. He was able to do this by constantly retraining his perceptual faculties to maximize the information gained from increasingly impoverished visual data, which led to him making new visual discoveries [44]. In an essay entitled ‘Perceptual systems, an inexhaustible reservoir of information and the importance of art’, he writes movingly of his pleasure and shock when studying a self-portrait by Pierre Bonnard for over an hour (far longer, incidentally, than is normally allowed for viewing stimuli in art-based psychology experiments). He said: ‘I think that art can be a unique, essentially non-verbal, channel of experience, by means of which one can access new and surprising experience and understanding of the infinite variety of reality.’ [45, p. 23].

Echoing these ideas, Bridget Riley, who has had a lifelong fascination with visual perception, writes: ‘We are all sometimes surprised by what we perceive, and this faculty can be trained, cultivated and developed to high levels.’ [42, p. 247]. Mann and Riley are among many artists who stress that intensive and prolonged perceptual engagement with the world and with artworks not only helps us to better understand what we do sense but reveals ever more to be sensed. Riley goes on: ‘The pleasures gained from looking at works of art and the world around us are inexhaustible. As Delacroix said, painting should be a feast for the eyes. That is a view to which I attach great importance. Looking is, I feel, a vital aspect of existence. Perception constitutes our awareness of what it is to be human, indeed what it is to be alive’ [42, p. 247].

Statements like these from prominent artists suggest that the process of subjecting reality to intense and prolonged scrutiny in order to make art that describes what is seen can draw us into another kind of paradox: we use the processes of active inference to learn more about the object we perceive—so tuning our generative model to the sensory data—while, at the same time, prediction errors about that same object proliferate the longer and more closely we study it. This conundrum is expressed by the painter Francis Bacon: ‘To me, the mystery of painting today is how can appearance be made…how can this thing be made so that you catch the mystery of appearance within the mystery of the making?’ [46, p. 105].

I take this opportunity to suggest that greater understanding of art and aesthetic affect will result from applying the PP framework in a way that accommodates both the paradoxical intuitions that pervade art thinking, and the rationalist aims of science. My tentative suggestion about how this might be achieved is, in brief, as follows.

According to PP theory, the purpose of perception is to guide a creature's behaviour using interoceptive and exteroceptive sensory data to maintain its optimal state and therefore preserve its existence [3]. Creatures like us do this by embodying a generative model that we use to infer the most probable causes of our sensory data, and by continually updating our inferences in light of new sensory stimulation. When there is a mismatch between the predictions made by the model and the sensory data it receives, a prediction error is generated. Probabilistic hierarchical processes attempt to minimize the error by updating the model or changing the sensory evidence through action.

As we have seen, powerful art experiences can induce high levels of prediction error. Indeed, they often produce states of mind that by their very nature are resistant to prediction error minimization because each of the conflicting hypotheses appears equally likely. Artists actively cultivate such states in themselves and aspire to evoke them in others through their art. So much so that this may be regarded, in behavioural terms, as seeking prediction error *maximization*, at least in the short term, while affording opportunities for its minimization in the long term. What might motivate such contrarian behaviour if the fundamental imperative of brain processing and bodily action is to minimize prediction error?

One possible response is suggested by the statement from Bridget Riley quoted above: ‘Looking is, I feel, a vital aspect of existence. Perception constitutes our awareness of what it is to be human, indeed what it is to be alive.’ If being aware is what it is to feel alive, and if highly salient prediction errors are marked by heightened state of awareness, then maximizing these errors, within certain bounds, can effectively heighten one's sense of existence. In other words, the multifarious, contradictory, paradoxical, and illogical states of mind produced in a person when they look intensely at the world, or at a powerful work of art, make us alive to the world.

To push this idea one step further, we might say that the beholder in the grip of the kinds of powerful aesthetic effects discussed here is experiencing what I have referred to elsewhere as the ‘fractured unity’ of consciousness. This is an oxymoronic condition in which the mind is at once divided and unified, and which is especially evident when appreciating art [47]. This state of mind may be pleasurable, where one resolves ambiguities and gains information through intense engagement with the subject in question, yet simultaneously bemusing, surprising, fascinating, and exhilarating because of the growing sense of mystery engendered by that same act of engagement. In an earlier work, *The Posthuman Manifesto*, I expressed it thus: ‘Rich aesthetic experience is generated by the perception, simultaneously, of continuity and discontinuity in the same event’ [48, p. 185]; where ‘continuity’ can be equivalent to ‘predictability’ and ‘discontinuity’ to ‘unpredictability’. But note that the bounds are also important, and the Goldilocks effect applies again: too little deviation from expectation, or too much continuity, is unstimulating; too much deviation, or discontinuity, threatens the agent's coherence and with it their continued sense of existence.

If these ideas capture something important about art experience, and PP aspires to explain such experience, then they would seem to invite further investigation using the tools and methods of the PP approach. Indeed, there are several promising ideas within the PP literature that might be applied. To consider only two: in a paper aimed at explaining the so-called ‘meta-problem’ of consciousness—that is, the problem of why we are puzzled by the fact of our own conscious experience—the authors argue that sufficiently sophisticated cognitive agents can generate counterfactual inferences from sensory evidence, even when they are completely certain about the reliability of that evidence, so leading to puzzlement [49]. This echoes some of the artistic experiences described above, where viewers find certain works mysterious even though aspects of the sensory evidence they evoke seems to be highly reliable, and this ‘drives a wedge between experience and the world’, as the authors cited above put it. And the fact that we seem to pay greater attention to ambiguous artworks may be because they require us to allocate ‘gain’ to some parts of the sensory input to increase the precision of the signal and thus its statistical weight in perceptual inference [50]. This activation of attentional mechanisms may in turn account for the heightened sense of awareness that Bridget Riley says we experience when looking intensely.

## 7. Conclusion

In this opinion piece I have explored the connections between artists ideas, art experiences and present-day scientific work in PP theory. Despite the different histories and ways of thinking, commonalities between them become apparent when we try to scientifically explain art experience using the PP approach, as the examples of the artworks discussed here, and the statements of other artists demonstrate.

I have also suggested that the insights gained from the scientific study of art using the PP approach can be applied to benefit our understanding of our perceiving minds and their relationship to our bodies and to the world. This further statement from *The Posthuman Manifesto* seems to be in sympathy with a core principle of PP concerning the role of perception for maintaining our biological integrity: ‘Since humans are continually faced with random stimuli, it is necessary to keep re-asserting order (maintaining meaning) so that we do not dissolve into chaos, thereby losing our sense of being.’ [48, p. 183]. But, as I hope I have shown here, merely being is not enough. The dissonant yet powerful experiences afforded by looking at certain art objects, and indeed by looking at the world in general, can intensify this sense of being in ways that give our lives the value and purpose that make them worth living. This might help us to understand some of the ‘mystery of appearance’ and what it means to exist at all.

## Data Availability

This article has no additional data.
